# Hand gestures for emergency situations: A video dataset based on words from Indian sign language

**DOI:** 10.1016/j.dib.2020.106016

**Published:** 2020-07-11

**Authors:** V. Adithya, R. Rajesh

**Affiliations:** Department of Computer Science, Central University of Kerala, Periya, Kasaragod, Kerala, India, 671320

**Keywords:** Indian sign language recognition, Hand gestures, Emergency words, Video data

## Abstract

Automatic sign language recognition provides better services to the deaf as it avoids the existing communication gap between them and the rest of the society. Hand gestures, the primary mode of sign language communication, plays a key role in improving sign language recognition. This article presents a video dataset of the hand gestures of Indian sign language (ISL) words used in emergency situations. The videos of eight ISL words have been collected from 26 individuals (including 12 males and 14 females) in the age group of 22 to 26 years with two samples from each individual in an indoor environment with normal lighting conditions. Such a video dataset is highly needed for automatic recognition of emergency situations from the sign language for the benefit of the deaf. The dataset is useful for the researchers working on vision based sign language recognition (SLR) as well as hand gesture recognition (HGR). Moreover, support vector machine based classification and deep learning based classification of the emergency gestures has been carried out and the base classification performance shows that the database can be used as a benchmarking dataset for developing novel and improved techniques for recognizing the hand gestures of emergency words in Indian sign language.

Specifications table

**Table:** 

Subject	Computer Vision and Pattern Recognition
Specific subject area	Automatic sign language recognition
Type of data	Videos
How data were acquired	The videos in this dataset were collected by asking the participants to stand comfortably behind a black colored board and present the hand gestures, in front of the board. A Sony cyber shot DSC-W810 digital camera with 20.1 mega pixel resolution has been used for capturing the videos.
Data format	Raw videos as well as cropped videos. The data are organized in two sets. One set contains the captured video sequences in original format (raw) and the other set contains the video sequences obtained after cropping out the excessive background objects, and downsampling the frames to a uniform size of 500x600 pixels.
Parameters for data collection	All the videos have been collected with plain black background by placing the camera at a fixed distance. Both male and female subjects from various parts of India with varying hand sizes and skin tones have been included for collecting the data. Two sample videos have been collected from each participant with the gap of small time duration. The data collection is done on different days and at different times in an indoor environment with normal lighting conditions. No restriction has been imposed on the speed of hand movements so as to get the gesture presentations as natural as possible.
Description of data collection	Videos for a set of eight hand gestures representing the ISL words namely, ‘accident’, ‘call’, ‘doctor’, ‘help’, ‘hot’, ‘lose’, ‘pain’ and ‘thief’ have been included in the dataset.
Data source location	Department of Computer Science, Central University of Kerala Periya, Kasaragod, Kerala India-671320
Data accessibility	Repository name: Mendeley data. Data identification number: DOI: 10.17632/2vfdm42337.1 Direct URL to data:https://data.mendeley.com/datasets/2vfdm42337/draft?a=c5c2265d-5dd2-4e67-8656-0af6527a9937

## Value of the data

The lack of publicly available dataset is a big challenge that hinders the developments in automatic SLR. The dataset proposed in this article is the first publicly available dataset of the hand gestures of the emergency ISL words. The data will be useful for the researchers to develop novel techniques for the improvements in automatic recognition of ISL gestures [1,2].
Improvement in this field is a great benefit to the society, as it provides a communication platform for the Deaf to convey their urgent messages to the authority.
This dataset can act as a basic benchmarking database of a set of hand gestures of emergency ISL words. It can be referred for further expanding the dataset by replicating the samples, or adding new samples of the gestures in different views and background conditions to further develop and improve the SLR and HGR techniques [3].

## Data description

The dataset contains the RGB videos of hand gestures of eight ISL words, namely, ‘accident’, ‘call’, ‘doctor’, ‘help’, ‘hot’, ‘lose’, ‘pain’ and ‘thief’ which are commonly used to convey messages or seek support during emergency situations. All the words included in this dataset except the word `doctor' are dynamic hand gestures. The videos were captured from 26 adult individuals including 12 males and 14 females in the age group of 22 years to 26 years. The subjects participated in the data collection process are not the representatives of a particular region, rather represent the whole India.

For dynamic gestures, hand gestures recognition depends most importantly on motion features, rather than skin color features, based on silhouette or shapes or edges and their variations over time due to its movements. Even though skin color variations play little role, the data collection has been done by taking extreme care to include participants with maximum skin color variations to study the dependency of gesture recognition performance on human skin color.

It may so happen that the skin color will certain time highly resemble to the background color (including person's clothing) and will highly affect the classification rate. Hence, all the videos in this dataset have been collected against a black background under normal lighting conditions in an indoor environment. Such type of black background can be easily constructed at a very low cost with a board painted in black color and placed in front of the camera. As these are emergency situation related words for use with the deaf to communicate with the world, high recognition rates with less false positive and less false negative are highly needed. The plain black color background in the videos helps to increase the performance of hand gesture recognition with less computational overhead.

**Fig. 1:**
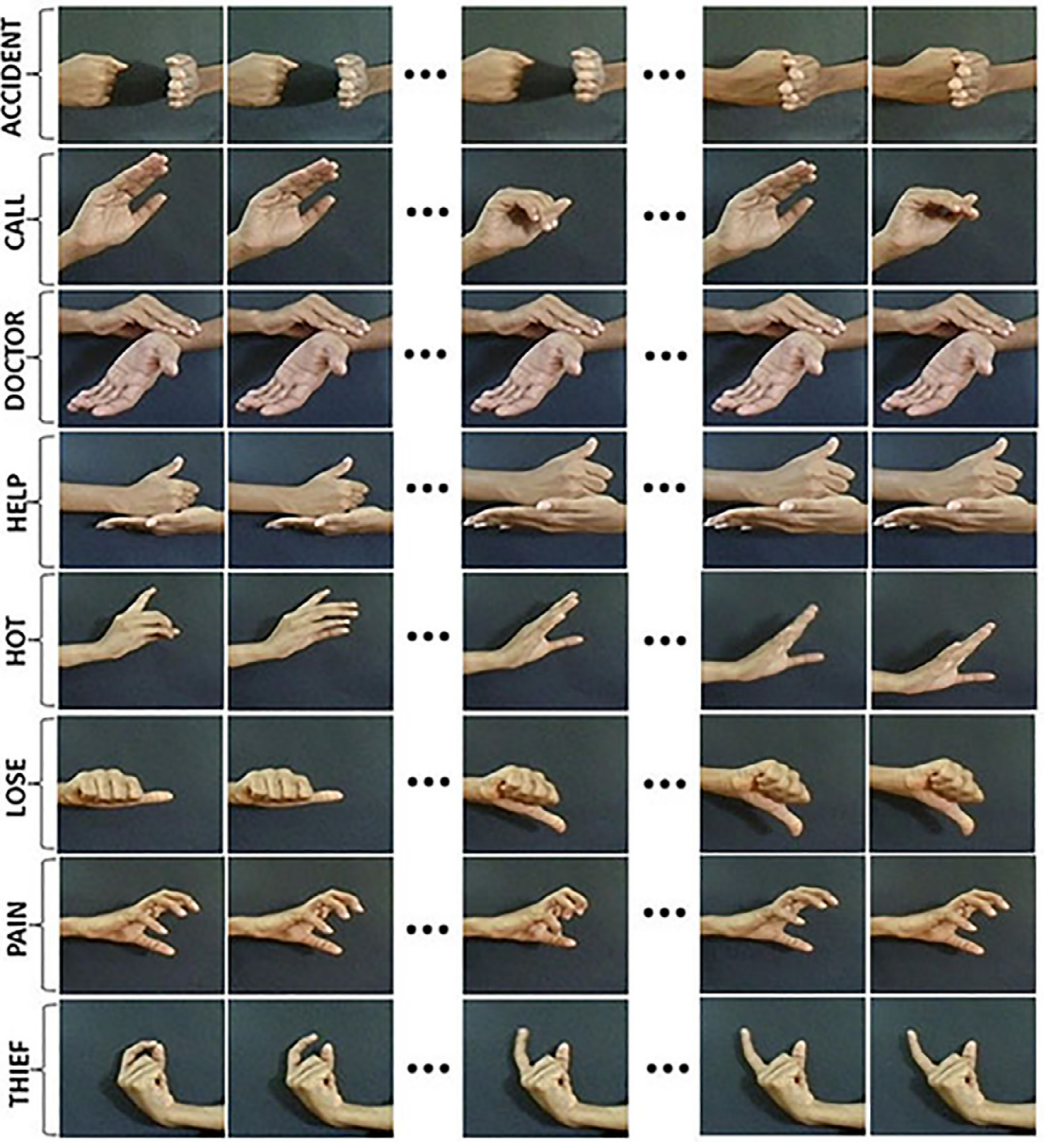
The key frame sequences of the hand gestures of the ISL words included in the `Cropped_Data’ set.

The dataset is built with an objective for developing a benchmark for emergency hand gesture recognition and the corresponding classification results as a reference for further improvements of the ISL recognition. The dataset is included in two folders namely ‘Raw_Data’ and ‘Cropped_Data’. The folder ‘Raw_Data’ contains the original ISL videos of size 1280x720 pixels. The folder ‘Cropped_Data’ contains the video sequences obtained after cropping out the excessive backgrounds and rescaling the frames to a uniform size of 500x600 pixels. Fig. 1 shows a set of keyframe sequences for sample videos from all the eight hand gestures in the ‘Cropped_Data’ set.

The dataset contains a total of 824 sample videos in *.avi* format. The raw videos are labelled using the format ***ISLword_XXX_YY***, where:

• ***ISLword*** corresponds to the words ‘accident’, ‘call’, ‘doctor’, ‘help’, ‘hot’, ‘lose’, ‘pain’ and ‘thief’.
• ***XXX*** is an identifier of the participant and is in the range of 001 to 026.
• ***YY*** corresponds to 01 or 02 that identifies the sample number for each subject.

For example, the file named ***accident_003_02*** is the video sequence of the second sample of the ISL gesture of the word ‘accident’ presented by the 3^rd^participant.

The cropped videos are labelled using the format ***ISLword_crop_XXX_YY***, where:

• ***ISLword*** corresponds to the words ‘accident’, ‘call’, ‘doctor’, ‘help’, ‘hot’, ‘lose’, ‘pain’ and ‘thief’.
• ***XXX*** is an identifier of the participant and is in the range of 001 to 026.
• ***YY*** corresponds to 01 or 02 that identifies the sample number for each subject.

For example, the file named ***accident_crop_003_02*** is the video sequence of the second sample of the ISL gesture of the word ‘accident’ presented by the 3^rd^ participant obtained after cropping and downsampling to 500x600 pixels. Table 1 and Table 2 shows the file and folder organizations of the sets containing raw data and cropped data respectively.

**Table 1:** Organization of raw videos in the dataset.

Folder	File Name	Description
accident_Raw	accident_001_01 to accident_026_01, accident_001_02 to accident_026_02	52 sample videos of ISL hand gestures for the word `accident' presented by 26 subjects.
call_Raw	call_001_01 to call_026_01, call_001_02 to call_026_02	52 sample videos of ISL hand gestures for the word `call' presented by 26 subjects.
doctor_Raw	doctor_001_01 to doctor_026_01, doctor_001_02 to doctor_026_02	52 sample videos of ISL hand gestures for the word `doctor' presented by 26 subjects.
help_Raw	help_001_01 to help_026_01, help_001_02 to help_026_02	52 sample videos of ISL hand gestures for the word `help' presented by 26 subjects.
hot_Raw	hot_001_01 to hot_026_01, hot_001_02 to hot_026_02	52 sample videos of ISL hand gestures for the word `hot' presented by 26 subjects.
lose_Raw	lose_001_01 to lose_018_01, lose_020_01 to lose_026_01, lose_001_02 to lose_018_02, lose_020_02 to lose_026_02	50 sample videos of ISL hand gestures for the word `lose' presented by 25 subjects.
pain_Raw	pain_001_01 to pain_026_01, pain_001_02 to pain_026_02	52 sample videos of ISL hand gestures for the word `pain' presented by 26 subjects.
thief_Raw	thief_001_01 to thief_019_01, thief_021_01 to thief_026_01, thief_001_02 to thief_019_02, thief_021_02 to thief_026_02	50 sample videos of ISL hand gestures for the word `thief' presented by 25 subjects.

**Table 2:** Organization of cropped videos in the dataset

Folder	File Name	Description
accident_Cropped	accident_crop_xxx_yy	52 sample videos of ISL hand gestures for the word `accident' presented by 26 subjects.
call_Cropped	call_crop_xxx_yy	52 sample videos of ISL hand gestures for the word `call' presented by 26 subjects.
doctor_Cropped	doctor_crop_xxx_yy	52 sample videos of ISL hand gestures for the word `doctor' presented by 26 subjects.
help_Cropped	help_crop_xxx_yy	52 sample videos of ISL hand gestures for the word `help' presented by 26 subjects.
hot_Cropped	hot_crop_xxx_yy	52 sample videos of ISL hand gestures for the word `hot' presented by 26 subjects.
lose_Cropped	lose_crop_xxx_yy	50 sample videos of ISL hand gestures for the word `lose' presented by 25 subjects.
pain_Cropped	pain_crop_xxx_yy	52 sample videos of ISL hand gestures for the word `pain' presented by 26 subjects.
thief_Cropped	thief_crop_xxx_yy	50 sample videos of ISL hand gestures for the word `thief' presented by 25 subjects.

## Experimental design

The hand gestures included in this dataset are according to the style and movements specified in the ISL dictionary published by the Ramakrishna Mission Vivekananda University, Coimbatore, Tamilnadu, India [4,5]. The videos of the ISL words and their descriptions have been shown to the participants for the effective presentation of the gestures. A Sony cyber shot DSC-W810 digital camera with 1280x720 pixels frame size is used for the data collection. This data collection process has got ethical clearance from Institutional Human Ethics Committee (IHEC) of Central University of Kerala, India. All the individuals have gone through the detailed informed consent form and signed their consent for voluntary participation.

**Fig. 2:**
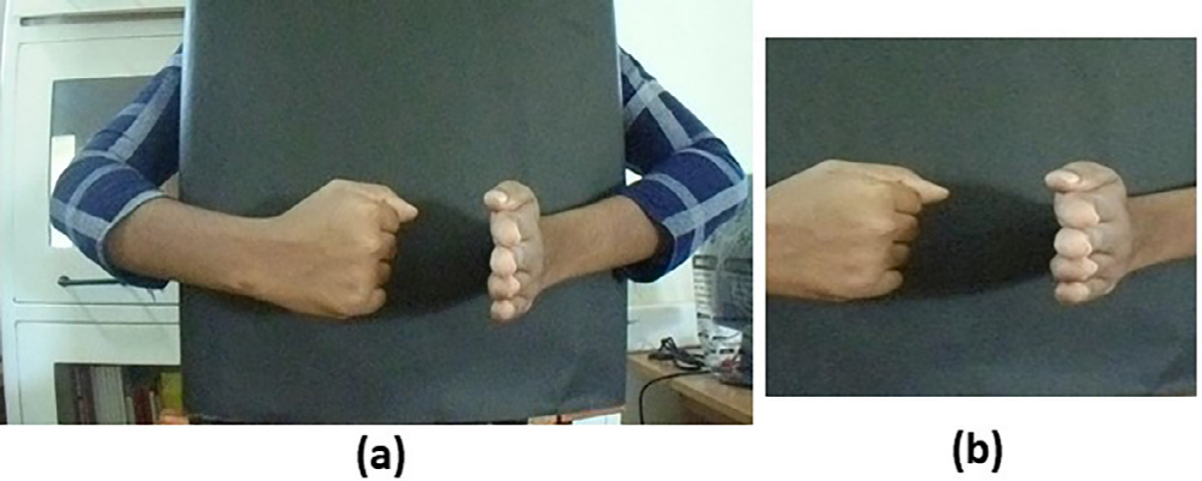
(a) A single frame of the video for the hand gesture of the word ‘accident’ in original form (b) the corresponding frame obtained after cropping and downsampling.

The videos in this dataset were collected by asking the participants to stand comfortably behind a black colored board. The participants were asked to present the eight hand gestures, in front of the board, one by one and the procedure is repeated two times to capture two sample videos of each gesture. Example for a single frame of the video of the word ‘accident’ in original form (raw) and after cropping are shown in Fig. 2(a) and Fig. 2(b) respectively.

All the videos were taken by fixing the camera at the same distance from the black board. Human hands are highly flexible in nature and the style and speed of hand movements by different individuals has shown great variations while presenting the gestures. No restriction has been imposed on the speed or time duration while capturing the video samples. Hence the duration of videos varies from one second to three seconds depending upon the speed of gesture presentations by different individuals.

## Data analysis

The ISL gestures in the cropped dataset have been analysed by classifying them with the conventional feature driven approach using multiclass support vector machine (SVM) [6] as well as the recently evolved data driven approach using deep learning model. In both cases, 50% of the dataset is used for training and the remaining 50% is used for testing.

In feature based approach, a set of key frames have been extracted from the video sequences through a fast and efficient method based on image entropy and density clustering as proposed in [7]. The keyframe extraction eliminates the redundant information and makes all the videos with equal numbers of frames. The appearance features, namely, the three dimensional wavelet transform descriptors [8] are extracted from the keyframes. These descriptors are used for training and testing the SVM classifier. SVM is a supervised machine learning approach used for binary and multiclass pattern recognition. The memory efficient operation through the data points called support vectors and the availability of versatile kernel functions make it a widely adopted choice for image/video based feature classification too. It is extensively used in classification problems with comparatively less training samples and shown greater performances. Multiclass SVM is utilized in this work and obtained an average classification accuracy of 90%.

In deep learning approach, the pre-trained convolutional neural network (CNN) model, namely GoogleNet [9], is combined with a long short term memory (LSTM) network [10] for gesture classification. The videos are converted into sequences of feature vectors through GoogleNet network, by getting the output of the activations of its last pooling layer. The classification model of LSTM network is built with a sequence input layer followed by a bidirectional LSTM layer with 2000 hidden units and a dropout layer afterwards. The output of the dropout layer is further transformed by the fully connected layer into the size suitable for classification by a softmax layer and a final classification layer. The network is trained for 20 epochs with the sequences of feature vectors in which 10% of the training dataset is used for validation with an adaptive moment estimation (adam) optimizer, a batch size of 16 and an initial learning rate of 0.0001. The performance of the classification model is evaluated with the test videos and achieved an average classification accuracy of 96.25 %.

The classification performances of both the methods have also been evaluated using the metrics for precision, recall and F-score values corresponding to each gesture class as shown in Table 3. The given average classification accuracies, precision, recall and the F-score values can be considered as the base performance measures and those who are further going to work on the dataset may improve it.

**Table 3:** Classification performance of multiclass SVM as well as deep learning model on the ISL words in the cropped set.

ISL Word	SVM Classifier			Deep Learning		
	Precision (%)	Recall (%)	F-score (%)	Precision (%)	Recall (%)	F-score (%)
Accident	96.55	93.33	94.92	100	100	100
Call	96.15	83.33	89.29	90.32	93.33	91.80
Doctor	90.63	96.67	93.55	93.75	100	96.77
Help	96.55	93.33	94.92	100	93.33	96.55
Hot	92.59	83.33	87.72	100	93.33	96.55
Lose	96.30	86.67	91.23	96.67	96.67	96.67
Pain	93.10	90	91.53	96.55	93.33	94.92
Thief	68.29	93.33	78.87	93.75	100	96.77

## Declaration of Competing Interest

The authors declare that they have no known competing financial interests or personal relationships which have, or could be perceived to have, influenced the work reported in this article.

## Appendix Supplementary materials

Image, application 1
